# Immigration Policy and Latinx/é Children from Mixed-Status Families: Mental Health Consequences and Recommendations for Mental Health Providers

**DOI:** 10.3390/children11111357

**Published:** 2024-11-08

**Authors:** Lucila Ramos-Sánchez, Jasmín D. Llamas

**Affiliations:** School of Education & Counseling Psychology, Santa Clara University, Santa Clara, CA 95053, USA; jllamas@scu.edu

**Keywords:** Latinx/é children, mental health, immigration

## Abstract

The impact of immigration policies on Latinx/é mixed-status families and their children is undeniable. Changes in immigration policy, focused on increased deportation enforcement, within the last three decades have led to an increased fear of deportation and unique circumstances mixed-status families must navigate. These circumstances, combined with fear of deportation, have had deleterious effects on the psychological well-being of the family, in general, and the children, in particular. This paper reviews the impact of immigration policies on Latinx/é mixed-status families, the unique circumstances of mixed-status families, and the mental health implications these have on the children specifically. Articles and books were selected from various sources that addressed Latinx/é mental health, mixed-status families, and immigration. After a review of the literature, these circumstances emerged: familial separation, citizen children second-class citizenry, developmental implications, psychological implications, and coping mechanisms and strategies of mixed-status families. Recommendations for mental health providers who work with children from mixed-status families are provided.

## 1. Introduction

As of 2022, immigrants with unauthorized immigration status grew from 10.5 in 2021 to 11 million [1]. This reversed a decade-long downward trend and was the first sustained increase since 2007. Mexico remains the most common country of birth, comprising 37% of the nation’s unauthorized immigrants, despite dropping by 2.9 million from 2007 to 2022. However, this decline was offset by the increase in the total number of unauthorized immigrants from countries other than Mexico. Three Central American countries (El Salvador, Honduras, and Guatemala) represent about 18% of the total unauthorized immigrant population, which saw a 50% increase in the last two decades [1]. This paper will focus primarily on the Latinx/é population as they constitute the largest percentage, approximately 55%, of unauthorized individuals. The term Latinx/é is used throughout the paper for gender inclusivity. Currently, there seems to be no clear consensus on which terms to use; therefore, both were used.

Unauthorized immigrants are those who reside in the United States without legal status, and this includes individuals who enter the country without inspection, who remain with an expired visa, and who are in a legalization process such as a spousal petition [1]. A consequence of unauthorized immigration has resulted in mixed-status families, which occur when the familial constellation consists of both U.S. citizens and unauthorized family members [2]. Almost 5% of all U.S. households have at least one unauthorized household member, with almost 70% of these households being mixed-status [1]. It is estimated that around 5.5 million children live in mixed-status families [3,4]. Of these children, 4.4 million were U.S.-born, and approximately 3.5 million were from unauthorized parents of Mexican descent [1,4]. Even though numbers may vary from year to year, the psychological impact remains consistent. Unauthorized Latinx/é immigrants encounter a myriad of stressors, but fear of deportation is a primary source of stress for many [5,6]. The fear has a profound impact on the Latinx/é community in general and the children from mixed-status families in particular.

The fear of deportation is warranted, especially given the changes in immigration policy that have resulted in increased deportations over the last three decades [7,8,9,10]. For context, a brief overview of shifting immigration policies is offered to illuminate the stressors of unauthorized Latinx/é mixed-status families and the impact on their children. One of the most significant changes to immigration policy leading to higher deportations was the continued expansion of deportation categories. Deportation categories are offenses that an unauthorized immigrant commits that are subject to deportation [11]. A conviction of an aggravated felony has always been subject to deportation in the U.S.; these included murder and drug trafficking [11]. Other lesser offenses that led to convictions were not subject to deportation. Immigrants with unauthorized status were often allowed to serve their time but stay in the U.S. However, in 1996, changes in immigration policy expanded categories of deportation to include any prison conviction of a year or more, irrespective of the crime [7]. Under former President Trump, the categories were further expanded to include any commitment to a chargeable offense irrespective of whether or not the offense was criminal and irrespective of conviction [12]. Therefore, a minor offense could lead to deportation. During the COVID-19 pandemic, several immigration restrictions were put in place, including Title 42, which was a public health law dating back to 1944 that was put into effect on the grounds of preventing the spread of COVID-19. Title 42 allowed U.S. officials to turn away migrants and deny migrants the right to seek asylum [13]. Title 42 was put in place in March 2020 and ended in May 2023. Even with this brief policy overview, it is clear how swift and constant immigration policy changes are.

Immigration policy directly dictates deportation rates. The rate of deportation was approximately 40,000 a year in 1996 and peaked in 2008 when deportation increased to 358,000 unauthorized immigrants a year [7]. The deportation rate from 2017 to 2019 remained consistently high: 226,119 in 2017, 256,085 in 2018, and 267,258 in 2019 [10]. The pandemic set in place several immigration restrictions that ultimately decreased the number of deportations, with large drop-offs observed from 2020 to 2022: 185,884 to 72,177, respectively [10]. This included the period Title 42 was in effect (March 2020 to May 2023), with approximately 2.8 million migrants turned away and denied the right to seek asylum [13]. By limiting the number of migrants processed, the number of deportations during this period decreased. Since the pandemic-era restrictions and Title 42 have been lifted, there has been a 97.5% (142,580) increase in deportations for 2023, with numbers seemingly on the rise [10].

The constant changes in policies and subsequent rates of deportation have led to significant fear and stress among unauthorized Latinx/é immigrants and their families [7,14,15,16,17]. In their annual report, ICE noted a concerted effort to prioritize resources to “stem irregular migration at the Southwest Border”, deploying more than 2500 officers and nearly doubling deportation numbers [10] (p. 2). Given the disproportionate effect of immigration policy on Latinx/é unauthorized immigrants, it is unsurprising this has a significant psychological impact on mixed-status Latinx/é families and their children.

Mental health providers may have a sense of immigration policy, the direct effects of deportation, and the subsequent impact on families and children; however, they may not be familiar with the specific and ongoing changes in immigration policies and practices. They may also be unaware of the specific circumstances mixed-status families encounter that specifically impact the children, as the literature in this area is still developing. The purpose of the paper is to highlight immigration policies that affect children at the local level, unique circumstances brought about by immigration policies and practices, and the implications these have on children from mixed-status families.

Children may be especially psychologically affected by the circumstances resulting from their parents’ vulnerable immigration status, as their coping mechanism to deal with intermittent and ongoing traumatic events may not be fully developed [18]. Therefore, mental health providers working with Latinx/é children from mixed-status families should be aware of the circumstances these children may experience on a daily basis. What follows is a summary of the unique circumstances Latinx/é mixed-statues families may encounter, how Latinx/é mixed-status families deal with potential familial separation, implications on the well-being of their children, and recommendations for mental health providers who serve the children.

## 2. Materials and Methods

In this systematic review, articles were primarily searched using the social science database APA PsycInfo. For immigration policy, Heinoline, Google Scholar, and Pew Research Center were used. The keywords used include immigration, Hispanic or Latino mental health and immigration, mixed-status families, and undocumented and mental health. The keyword Hispanic was used rather than Latiné because the term Hispanic or Latino was formerly used throughout the literature. More recent research used some variation of Latiné.

Articles and books were accessed between the years 1998 and 2024 to capture the impact of immigration policy changes. Works included articles and books rather than just research articles because this area of research is still developing. Both English and Spanish articles were reviewed; however, most were in English. The review did not limit or exclude articles based on study design as long as the study met the criteria for relevant content related to Latiné mental health, mixed-status families, and immigration. Articles and books were initially screened by title, then abstract review, followed by evaluation of the entire article or chapter. The literature was evaluated, and the information extracted was authors, year, and findings. This search resulted in 78 articles and books. From these, 63 total articles (53), reports (5), and books (5) were included. Fifteen were excluded from the final review. Articles were not included in the review if they were tangentially related to immigration policy and the mental health of mixed-status families and their children. For example, some articles focused on health care access for undocumented individuals or adjustment in their home country because of deportation. The articles were original research and published in peer-reviewed quality journals. Reports were included in order to provide recent demographic information. No software or data management programs were used. Through the evaluation of research articles and the literature, five themes emerged for children in mixed-status families. These are presented in the following section. There is a dearth of research in this area with substantial gaps. As such, the literature extended beyond research articles to include books and conceptual works.

## 3. Circumstances of Latinx/é Mixed-Status Families

### 3.1. Familial Separation

Mixed-status families are at high risk of familial separation because of the possibility of detainment or deportation [10,19,20,21,22]. There are numerous constellations of mixed-status families that include but are not limited to one or both parents being unauthorized with U.S. citizen children or one or both parents being unauthorized with children of different citizen status. Detainment or deportation of an unauthorized family member may result in different decisions or considerations depending on the constellation of the mixed-status family.

When unauthorized parents with citizen children are detained or are served with deportation orders, parents must make the excruciating and stressful decision of whether to relocate with their U.S. citizen children or leave their children in the U.S. [23,24]. This is referred to as the deportability of U.S. citizen children [24]. If parents choose to return to their home country with the entire family to avoid separation, the citizen children will be forced to live in an unfamiliar country [25]. The children may not know the language or be familiar with the culture, having been socialized in the U.S. This may be particularly hard on U.S. citizen children or adolescents who have acculturated to U.S. customs and norms because of their participation in the K-12 education system [26,27]. Furthermore, citizen children who relocated with their unauthorized parents suffered higher rates of depression than citizen children whose parents were not impacted by detention or deportation [24]. Though families may choose to live in countries in which they are unfamiliar or do not know the language, they may do so by choice rather than forced removal, which may be more stressful. In fact, involuntary migration is known to be substantially more stressful and traumatic than voluntary migration [21].

If only one unauthorized parent is deported or detained, the family must decide whether to join the deported parent or to stay in the U.S. Assuming one parent is able to stay in the U.S., the separation results in a single-parent household, which may lead to trauma, economic hardship, and increased fear of deportation [7,22,27,28]. Men were deported at a much higher rate compared to their female counterparts [29,30]. This is referred to as the gendered process of deportation, which often resulted in single-parent households primarily headed by unauthorized women [31]. The change to a single head of household increased poverty and economic hardship, especially for unauthorized Latinx/é women who earn substantially less than their authorized counterparts [32]. The lower earning potential and the loss of a second income for many unauthorized women resulted in defaulting on bills, mounting debt, housing instability, and food insecurity [33]. Research has found similar findings for families of detained individuals who experienced extreme financial insecurity and an inability to pay their mortgage or monthly bills [34]. Researchers estimated that within 6 months, families lose 40–90% of their income, or an average of 70% [35].

In response to a decrease in income, older children often need to take on jobs to help support the family, impacting their school performance, persistence, and retention [33]. Additionally, if the mother is working extended hours and multiple jobs, older children may also be tasked to serve the role of primary caregivers to younger siblings due to the lack of affordable childcare options. Children, especially older children, burdened with financial and caregiver responsibilities, can experience stress that impacts their school performance, persistence, and retention while reducing emotional connection with the remaining parent. Sudden separation can greatly impact the parent–child relationship depending on how the older children respond to the new responsibilities [30,36].

The family unit may also become fractured due to deportation when both unauthorized parents are deported but choose to leave behind their U.S. citizen children [23]. Some unauthorized Latinx/é parents may decide they do not want to socially uproot their children. Others may leave their children in the U.S. so the children can take advantage of the economic and academic opportunities not available to them in their parent’s home country. Children may be left with a trusted family member or friend. In some instances, they may be left with the oldest sibling if the citizen sibling is of age [23]. In these cases, this places undue financial, social, and emotional responsibility on the oldest citizen sibling.

### 3.2. Citizen Children Second-Class Citizenry

Citizen children may suffer consequences because of their parents’ documentation status, which is similar to their unauthorized parents. Children born in the U.S. have legal rights but may not enjoy the full extent of their rights because of their parent’s immigration status. This led to what is referred to as second-class citizenry when citizen children’s rights as U.S. citizens were not respected [37]. A study of multigenerational punishment found that the impact of immigration laws extended beyond the intended unauthorized Latinx/é parent to their citizen children [37]. Multigenerational punishment manifested because citizen children also experienced their parent’s fears; they sought to lessen their parent’s concerns, and they, too, feared deportation. Similar to their unauthorized parents, citizen children reported adopting hypervigilance and fear of police as they aged [37]. Therefore, they, too, were vulnerable to the ever-changing immigration policy, especially during the Trump administration. The former President announced a desire to end birthright citizenship, further jeopardizing the rights of children of unauthorized parents. These children, despite legally having the same freedoms as other citizens, are subject to the same uncertainty over immigration policy, fears of government, and barriers as their unauthorized family members [38].

Citizen children of unauthorized parents experienced food insecurity, economic insecurity, limited transportation mobility, limited travel opportunities, limited social friend groups, and vigilance similar to their parents [39,40]. Economic insecurity had significant broad, long-term implications for multigenerational punishment and second-class citizenry, which included less developed social capital and cultural capital, diminished educational opportunities, and limited upward social mobility. Researchers concluded that this second-class citizenry reproduced inequities in subsequent generations [37]. Similarly, another study found that parent’s vulnerable immigration status was detrimental to the social integration of their citizen children [41]. Not surprisingly, a parent’s vulnerable documentation status was related to an increase in their children’s anxiety, aggression, depression, and isolation [30,42,43,44,45,46].

### 3.3. Development Implications

In mixed-status families, the parent’s documentation status can have implications on their children’s development because of the limited access to early education and health services [30,36,42,47,48]. Even though citizen children are entitled to these programs and services (e.g., Medicare, public housing, and food stamps) that would benefit the whole family, unauthorized parents may not access services for their children out of concern about revealing their immigration status. Additionally, given the constant uncertainty over what changes new administrations will make to immigration policies, mixed-status families can be wary of government services. In some instances, public agencies may use “scare tactics” such as asking parents for identification, which unauthorized parents may not possess [47]. This may explain why studies have found that children of unauthorized parents were not enrolled in early childhood education programs such as preschool or other developmental enriching programs compared to children of authorized parents [39,40,49,50,51,52,53,54]. Lack of access is particularly detrimental as it comes at a critical time in a child’s cognitive development [48].

### 3.4. Psychological Implications

Parents’ vulnerable immigration status can have implications on their citizen children’s well-being even if parents have not been involved in deportation proceedings. Often, unauthorized parents’ constant worry and concern were transmitted to their children and affected their children’s mental health [54]. In fact, children from mixed-status families frequently expressed high levels of stress because of the uncertainty in their daily lives [43]. Other researchers have found high levels of anxiety resulting from fear of losing an unauthorized parent, whether or not the parent was under removal proceedings [24]. The anxiety because of deportation can often be felt throughout the community. This likely occurs because, for every two adults who were deported, one citizen child was directly affected, irrespective of whether the adult was in their household [55].

For children with direct experience of parental separation due to deportation, the psychological impacts are devastating [46]. Between the loss of their parent or caregiver, fears around family stability, and fears for their own safety, deportations can be a double or even triple trauma for young people [56]. Family separation due to deportation has a negative impact on the child’s mental health, psychosocial and academic outcomes, and well-being [57]. Children who experience parental separation may exhibit depression, nightmares, eating problems, school failures, and other somatic complaints [20,21]. Other studies have found that Latinx/é children whose parents were either detained or deported became socially withdrawn, fearful, and anxious [24,30,33,42,50]. Additional research has found children with a parent who had been deported were more likely to display externalizing (e.g., aggression, conduct problems) and internalizing (e.g., anxiety, depression) problems [58]. The deleterious effects are profound and long-lasting. Some research has described the loss of a parent to deportation as an “ambiguous loss” because the absence is neither permanent nor definitive, with uncertainty around when and/or if they will ever be reunited [59,60]. The uncertainty exacerbates anxiety and stress, leaving the child with unresolved grief, never knowing if they will ever reunite with their parent(s) again [56]. As a result, there is a significant need for mental health providers to become familiar with the experiences and circumstances of citizen children and noncitizen children left behind after familial separation due to parental deportation. Equally as important is having familiarity with the coping strategies parents may have in the event of deportation.

### 3.5. Coping Mechanisms and Strategies of Some Mixed-Status Families

The possibility of deportation has led many mixed-status families to develop coping mechanisms and strategies to deal with their circumstances [37]. Some families engaged in impression management or try to “pass” as documented individuals. Unauthorized individuals reported driving a newer car because police were more likely to profile older cars. Unauthorized parents reported discussing the threat of deportation with their children and discussing a contingency plan in the event the unauthorized parent was detained or deported. This assumes unauthorized parents have time to make these decisions if they are detained. In many instances, unauthorized individuals may be deported without due process or judicial review [61].

Examples of contingency plans may include making prior arrangements with their children as to who will stay in the U.S. and who will return to the country of origin [37]. This often depends on whether one or both parents are deported. In some instances, the entire family may return, depending on the age of the children. If only one parent is detained, the other may stay with the children. In the event both parents are detained or deported, families may identify a designated caretaker with whom to leave their children. If one of the children is of age, parents may leave the younger children in the care of the oldest child. Other families may identify a close neighbor, family friend, or relative as a designated caretaker in the event the parents are deported. Children were instructed on where to go if their parents did not pick them up from school, such as contacting a predetermined designated adult [37]. The assumption is that if parents fail to pick up their children from school or do not come home from work, then it is possible the parents were detained by immigration officials. Many families find it necessary to prepare for the threat of deportation well before it occurs. Even though these conversations may be necessary, they may increase trauma, fear, and anxiety in their children.

## 4. Discussion

The research on mixed-status families elucidates the impact of parents’ documentation status on the mental health, development, daily functioning, and second-class citizenry of citizen children. Of course, this is exacerbated if the children are unauthorized as well. Mental health providers who work with the Latinx/é population, and specifically Latinx/é children from mixed-status families, should be mindful of these stressors and how these stressors may impact children’s overall functioning. With this in mind, the following recommendations are offered to help facilitate and guide work with Latinx/é children from mixed-status families.

Providing culturally responsive and culturally informed mental health services is essential. Offering mental health services within the cultural lens of the clients can help the mental health providers determine an appropriate culturally relevant treatment plan and/or intervention. Agencies can ensure sufficient bilingual/bicultural mental health providers are available to work with the children and their families. There may be instances where Latinx/é children are fluent in English, but their parents may not be fluent in English at the same level. Bilingual mental health providers are needed to ensure they can communicate and consult with monolingual Spanish-speaking parents about their children.

Children are particularly vulnerable to the consequences of parental deportation [48,55]. Children who have lived through a parent’s deportation may experience trauma, especially if they witnessed the event. Moreover, if they continue to hear about deportations occurring around them in the community, Latinx/é children may be retriggered, making healing difficult. Continually being in a community where deportations occur can have severe implications for the mental health of Latinx/é children, which may result in continuous trauma. Therefore, mental health providers are encouraged to develop their ability to work with children presenting with trauma or symptoms of trauma.

Any mental health providers working with children from mixed-status families are encouraged to learn about immigration policies and laws that impact mixed-status families and their children. This paper provided a brief overview of immigration laws and policies; however, these laws and policies have shifted and expanded quickly. Therefore, staying up-to-date is essential in understanding how these ongoing changes impact the daily functioning of mixed-status families and the subsequent impact on the children. This is particularly important given the change in presidential leadership. Mental health providers are encouraged to advocate for immigration policies that reduce familial separation and stress related to unauthorized status. This may seem outside the scope of counseling; however, it is imperative mental health providers use their privilege to advocate for individuals who cannot advocate for themselves.

In instances where parents develop a deportation contingency plan if a designated caregiver has been identified, mental health providers may want to recommend parents complete a designated caregiver form. Brooklyn Community Foundation referred to the form as the “Designation of a Person in Parental Relation [62]”. The Foundation suggests identifying who will be the designated caretaker, informing the person they select, completing the form, and leaving a copy with the designated caretaker in the event deportation happens quickly. The form authorizes the child’s doctors, teachers, and school to share confidential information about the child with the caregiver. The Foundation further recommends any important documents be copied and shared with the designated caregiver as part of the planning process in the event of deportation.

Mental health providers are encouraged to learn about resources or develop a knowledge base that may benefit mixed-status families and their children. One such knowledge base is immigrants’ rights as it relates to unauthorized status. Mental health providers can make available brochures and websites that inform mixed-status families of their rights. Partnering with community-based organizations, having a variety of legal resources such as law clinics, and having contact information for immigration attorneys could help guide families in crisis. Another resource is the American Civil Liberties Union, which is one of the leading resources of immigrant rights [63]. The ACLU provides information on the rights of unauthorized immigrants residing in the U.S., what the person should do if they are stopped and questioned by police or immigration officials, what people should do if immigration officials or police arrive at their home, and the ACLU outlines a person’s rights if they are detained. Having these resources can help reduce parental stress and, by extension, benefit their children.

Becoming familiar with social service resources beneficial to children of mixed-status families is also necessary if mental health providers are to be effective with this population. As noted previously, parents with unauthorized status may fear applying for social services their children are entitled to as citizens of the U.S. Therefore, familiarity with the application processes in order to inform the parents could help alleviate parental concerns. For example, knowing what is asked of the parents, what documentation is required, and how long the process will take are good first steps. Mental health providers may also want to establish professional relationships with individuals who administer these services so that the referral is made to a known, trusted person. This can help reassure the parent with unauthorized status, and children can receive the services they need.

Staying abreast of what is happening in the Latinx/é community with regard to detainment and deportation may help anticipate the duress children from mixed-status families may be experiencing. For example, mental health providers should be aware of deportation and ICE activity in the area. Deportations and ICE activity vary across the country and even across the state, which may ebb and flow depending on the region. Staying in touch with community organizations, immigrants’ rights groups in the community, and local consulates may be a good source of local deportation information. Another source is local news, which may be a better resource than national news because national news may not be a good indicator of what is happening at the local level.

Finally, given current events, the responsibility of mental health providers extends beyond helping clients one-on-one. The social justice orientation within the profession of psychology suggests mental health providers take an advocacy role for vulnerable populations that go beyond the therapeutic office. Mental health providers can appeal to their local politicians to establish certain spaces in their city and/or local municipality as designated safe spaces or sanctuaries. These could include buildings or agencies (building of a non-profit agency or a church) where unauthorized individuals and their families can seek refuge. A similar designation could be afforded to an entire city so it can be considered a sanctuary city. A sanctuary city has laws to protect unauthorized individuals from deportation and prosecution, irrespective of federal law [64]. This discourages cooperation of local law enforcement and other government agencies or entities to partner with Customs and Immigration Enforcement (ICE) officials. Mental health providers can call or email their representative and similarly advocate keeping mixed-status families together with their children in the U.S. Joining professional associations that have a history of proposing policies and advocating for unauthorized individuals so they are not separated from their children is another path mental health providers can follow. Finally, considering the effects on citizen children, mental health providers can advocate for the rights of citizen children so they are protected or are ensured services they are entitled to in the U.S. Interventions can also be developed in the community that help inform and connect families to the services for their citizen children. In this way, they may serve as a bridge to the services to increase access. In essence, the mental health profession can and should play a larger part in promoting the advancement of rights and fair treatment of others.

## 5. Conclusions

The ongoing consequences of Latinx/é parents’ documentation status can have profound effects on their children. Along with the usual developmental, social, and psychological factors children must navigate, Latinx/é children of mixed-status families encounter additional stressors that can have long-term effects on their overall well-being. Mental health providers can play a vital role in helping Latinx/é children from mixed-status families cope with the ongoing environmental stress that may affect their mental health and everyday lives. The recommendations outlined in this paper encourage mental health providers to engage in continuing education about immigration policies, relevant resources, and local events. This may go beyond traditional interpersonal therapy and interventions, but it may be necessary to work effectively with children from mixed-status families.

## 6. Future Directions

The existing literature highlights the psychological implications of parental immigration status on their children. However, more research is needed to elucidate the social context of citizen children who must care for younger siblings and the struggles they encounter. Little is known about their daily lives, the impact on their mental health, and how they navigate social services. This research could inform how interventions and services are tailored to address their specific needs. Future research can also focus on the implications of the inability to integrate into society stemming from parental unauthorized status. Societal exclusion may have generational effects that may not be evident because they are structural in nature. Finally, few studies have examined familial dynamics and stress associated with having children with different immigration statuses. Future studies could examine how different immigration statuses influence expectations and responsibilities within the family and how immigration status impacts children’s educational aspirations. Overall, more research is needed in these areas so mental health providers can offer support and interventions and better target the needs of children from mixed-status families, as there seems to be no decline in immigration and the impact on Latinx/é children.
